# On the Periodicity of Neuroses

**Published:** 1845-12

**Authors:** J. Pidduck

**Affiliations:** London


*On the Periodicity of Neuroses.* By J. Pidduck, Esq., M.D., London.—The periodical recurrence of the paroxysms of nervous diseases is a fact not less interesting in a practical, than it is instructive in a pathological, point of view. It necessarily divides the treatment into two parts—that which is to be employed during the paroxysm, and that which is to be employed in the intervals.

The case of Mary Ann H—— is adduced, as a singular instance of a neuralgic periodicity, and of apparently successful treatment. The case is singular, both from the equal length of the paroxysms and of the intervals, as well as from the regularity of their recurrence.

Her age is fourteen. Catamenia have not appeared. Six years ago she was thrown out of a swing, and her head struck against a brick. Violent headache, referred to the forehead, ensued, which lasted about a fortnight; the right hand then became clenched, and the headache ceased. The hand continued immovably closed for three weeks; it then opened of its own accord, and the headache returned. The hand remained open, and she had the free use of it for three weeks, during which time she suffered from headache. It then closed, with the same relief from headache, which she at first experienced. Besides the freedom from headache, her general health was invariably better when the hand was closed, and the opening of the hand was invariably preceded by feelings of indisposition.

From the time of the accident to August last, a period of six years, the alternations of closing and opening of the hand and of headache have followed each other with perfect regularity, except sometimes during the summer, when the intervals have been somewhat protracted. Once, in summer, the hand remained open two months, with constant fore-headache. Instances of injury sustained by the upper part of the spine, from the *contre-coup*, in which the pain is referred to the forehead, are common.

Two cases of this kind have come under my care, attended with incessant vomiting so long as any food remained in the stomach. The first was of a boy, who fell, head foremost, on the pavement, in leaping over a post. The pain was referred to the forehead, where there was a slight bruise. The tenderness, on pressure, was in the upper cervical vertebræ. The pain in the forehead, the vomiting, and the tenderness, were removed by leeching the upper part of the spine, and by rest in the horizontal position.

The second was of a young man, who was seriously hurt by the falling of a heavy wheel on his back, when he was stooping. The pain in the forehead, continual vomiting, and tenderness in the atlantic region, were removed by the same treatment—repeated leeching, and rest in the recumbent posture.

When Mary Ann H—— came under my care in August last, the hand was closed, and she made no complaint of headache. A forcible attempt made to open the hand gave her great pain, extending up the arm. I was struck with the excessively sour smell of the perspiration in the hand, resembling sour curds, just as putrefaction is commencing—a state of the cutaneous secretion which obtains in this form of nervous affections. She complained of tenderness along the cervical vertebræ. A grain of strychnia, divided into fifteen pills, one to be taken three times a day, was prescribed, and a succession of mustard-poultices along the spine. I directed the hand to be well washed with soap and water, and ordered an antacid diet. Under this plan of treatment her general health underwent a manifest improvement, and when the hand opened at the expiration of the last three weeks of its closure, she ceased to complain of her head, which she had never done on former occasions.

The strychnia, which had been continued fifteen days was omitted when the hand opened, and the decoction of polygala senega, with sulphate of magnesia, was substituted, with the use of the vapor bath. These means were used for a month. Finding there was no return of closure of the hand nor of headach, that the tenderness of the cervical vertebræ was no longer felt, and that her health in every respect was good, the remedies were discontinued, and she has remained perfectly well. This case exhibits the utility of strychnia in small doses as an anti-neuralgic medicine. Six cases, showing its successful operation, were published by me in the *Medical Gazette.*

But the chief object in relating the case of Mary Ann H— was, to show a singular instance of periodical nervous affection. If it were not for the *medicina perturbatrix*, generally resorted to in diseases of the nervous system, I believe that their periodical character would be much more frequently manifested, from the most violent mania to an ordinary toothache. The term lunacy, anciently applied to the former, and face or brow ague, when the pain was reflected from the exposed nerve of a carious tooth, along some superficial branch of the fifth pair of nerves, to the latter, may be traced to the popular observation of their periodical nature.

The question arises:—How can the extraordinary regularity of the paroxysms, in the case in point, be explained?—three weeks of head affection, with perfect use of the hand, and three weeks of rigid closure of the hand, with perfect freedom from head affection, and that for a term of six years! The only explanation that can be offered is perfectly hypothetical, as every explanation of the morbid phenomena of the nervous system must be, so long as we remain ignorant of the nature, properties, and mode of distribution of the nervous energy. The same law of the animal economy which regulates the circulation of the blood seems to determine the distribution of the nervous energy. The same causes which disturb the equality of the circulation also disarrange the nervous influence. By the fall on the head, the upper part of the spine received the principal shock, or *contre-coup,* evidenced by the tenderness in that part. The circulation and innervation were disturbed, an accumulation of blood and nervous energy, or plethora, in the cervical portion of the spinal cord, ensued, which was periodically relieved by the spasmodic closure of the hand. That the vapor-bath was of service in promoting an equal distribution, both of blood and of nervous energy, after the last paroxysm, I think must be admitted; and that to its use the prevention of the recurrence of the closure of the hand must be ascribed.

The *modus operandi* of the strychnia, like that of the galvanic and electric fluid, probably is by exhausting the nervous energy, and thus promoting the relaxation of the spastic rigidity; for, in cases of tonic and clonic spasm, or *distentio nervorum*, these medicinal agents are more successful than in cases of paralysis, or *resolutio nervorum.* This case also shows the value of the vapor-bath as a remedy in neuralgia, and the importance of an antacid diet; for, in the treatment of these frequently intractable cases, every remedy has been unsuccessful, unless a total abstinence from fruit, wine, beer, vinegar, and vegetable acids of all kinds has been strictly observed.—*Ibid.*

